# Effect of γ-secretase inhibitor on Th17 cell differentiation and function of mouse psoriasis-like skin inflammation

**DOI:** 10.1186/s12967-018-1442-6

**Published:** 2018-03-10

**Authors:** Lei Ma, Haibo Xue, Ruiqun Qi, Yanqin Wang, Libing Yuan

**Affiliations:** 1 Department of Dermatology, Binzhou Medical University Hospital, 661 Second Huanghe Road, Binzhou, 256603 China; 2 Department of Endocrinology and Metabolism, Binzhou Medical University Hospital, 661 Second Huanghe Road, Binzhou, 256603 China; 3 Department of Dermatology, The First Affiliated Hospital of China Medical University, 155 North Nanjing Road, Shenyang, 110001 China

**Keywords:** Psoriasis, Notch1 signaling, Th17 cells, γ-secretase inhibitor

## Abstract

**Background:**

Th17 cells and its effective cytokine IL-17A play an important role in the pathogenesis of abnormal immune responses in psoriasis. Notch1 signaling has been implicated in Th17 cell differentiation and function. In this study, our aim was to evaluate the possible inhibitory effect of Notch1 signaling inhibitor, γ-secretase inhibitor DAPT, on psoriatic Th17 cell differentiation and function in a mouse model of psoriasis-like skin inflammation.

**Methods:**

Mouse psoriasis-like skin inflammation model was established by topical 5% imiquimod (IMQ) application, and experimental mice were divided into control group, IMQ-treated group and IM + DAPT-treated group. DAPT and the equivalent amount of Dimethyl sulfoxide was intraperitoneally injected in IMQ + DAPT-treated group and the other two experimental groups respectively. Skin tissues of the three experimental groups were acquired and stained with haematoxylin and eosin (HE). Splenic single-cells and serum were collected to detect the percentage of Th17 cells, the mRNA expression levels of Notch1 and its target gene Hes-1, Th17-specific transcription factor RORγt and its effective cytokines IL-17A, as well as IL-17A serum concentration. In addition, splenic CD4^+^ T cells from IMQ-treated mice were isolated and treated by DAPT to further measure the inhibitory effect of DAPT on the Th17 cell differentiation and IL-17A secretion in vitro.

**Results:**

DAPT treatment alleviated the severity of IMQ-induced mouse psoriasis-like skin inflammation and decreased the scores of erythema, scaling and thickening. HE stain reveals obviously reduced epidermal hyperplasia and dermal inflammatory cells infiltration in IMQ + DAPT-treated mice. The increased expression of splenic Th17 cell percentage, along with Notch1, Hes-1, RORγt and IL-17A mRNA and IL-17A serum concentration in IMQ-treated mice were significantly decreased when experimental mice were treated by IMQ and DAPT combinedly. Data obtained from in vitro study in IMQ-treated mice also demonstrated that blocking Notch1 signaling by DAPT can result in a dose-dependent decrease of Th17 cell proportion, mRNA expression of Notch1, Hes-1, RORγt and IL-17A as well as IL-17A secretion in splenic CD4^+^ T cells.

**Conclusion:**

These data suggest that Notch1 inhibition by DAPT can effectively alleviate the severity of mouse psoriasis-like skin inflammation by regulating the differentiation and function of Th17 cells, indicating that DAPT might be a potential therapeutic candidate for the treatment of psoriatic inflammation.

## Background

Psoriasis is a common chronic, immune-mediated, systemic inflammatory disease [1–4]. Psoriatic lesion is characterized by keratinocyte hyper-proliferation with activated CD4^+^ lymphocytes and neutrophils infiltration in the dermis [5]. The infiltrated lymphocytes in psoriatic lesion have been traditionally considered to be comprised only by Th1 lineage [6]. Recently, a distinct subtypes of CD4^+^ T cell, Th17 cells, have emerged as a key player in psoriasis pathogenesis, which are more highly expressed in psoriatic dermis and make up more than 50%, even 90% of the CD4^+^ population in psoriatic lesions [7–9]. RORγt is the specific transcription factor of Th17 cells, which is essential for Th17 development and function [10]. IL-17A, the principal effective cytokine of Th17 cells, functions as a proinflammatory cytokine, which up-regulates a number of chemokines and leads to the recruitment of neutrophils into sites of inflammation [11]. Numerous studies have shown an increased expression of Th17 cells and IL-17A in psoriatic lesion and peripheral circulation and associated with the disease severity [8, 12–15]. Therefore, suppressing Th17 response may be an effective therapeutic strategy for treating psoriasis.

Notch signaling is involved in a broad spectrum of cellular activities such as differentiation, proliferation, and regulation of function, including early T cell development in the thymus and modulation of peripheral T cell differentiation [16–19]. Notch signaling is initiated when Notch receptors are engaged with a Notch ligand, then a series of enzymatic reactions result in the cleavage of the Notch receptor intracellular domain (NICD), which is then translocated to the nucleus and initiates the transcription of downstream genes, such as Hairy/enhancer of split-like 1 (Hes-1). The effect and function of Notch signaling can be effectively blocked by γ-secretase inhibitors through inhibiting active NICD release. Notch1 signaling has been demonstrated to be crucial in both mouse and human Th17 cell differentiation. Blockade of Notch1 signaling can result in markedly down-regulated expression and secretion of IL-17A and prevented the progression of Th17-mediated disease, such as experimental autoimmune encephalomyelitis [20].

Up-regulated expression of Notch1 has been demonstrated in psoriatic lesions, which indicates that Notch1 signaling may participate in the pathogenesis of psoriasis [21]. In this study, we aimed to evaluate the possible inhibitory effect of γ-secretase inhibitor *N*-[*N*-(3,5-difluorophenacetyl)-l-alanyl]-*S*-phenylglycine t-butylester (DAPT) on Th17 cell differentiation and function in a mouse model of psoriasis-like skin inflammation.

## Methods

### Mice and treatments

BALB/c mice, 6–8 weeks old, weighting 18 ± 2 g, were purchased from Beijing Vital River Laboratory Animal Technology Company (Beijing, China) and bred in specific pathogen-free environment in the animal center of Binzhou Medical University Hospital. Mouse psoriasis-like skin inflammation was established as described previously [22]. Experimental mice were divided into three groups: control mice (n = 10), IMQ-treated mice (n = 18) and IMQ + DAPT-treated mice (n = 10). Control mice were treated with vaseline cream simply on the shaved back every day. IMQ-treated mice received a daily topical dose of 50 mg/cm^2^ of commercially available 5% imiquimod (IMQ) cream (Zhuhai Federal Pharmaceutical Company, Zhuhai, China). In IMQ + DAPT-treated mice, DAPT (Sigma-Aldrich company St Louis, MO, USA) was prepared by dissolving in Dimethyl sulfoxide (DMSO, Sigma-Aldrich) and intraperitoneally injected (10 mg/kg/day) since the beginning of IMQ application. Control mice and IMQ-treated mice received the same amount of DMSO. The changes of skin structural characters were observed and objective scores based on the clinical Psoriasis Area and Severity Index (PASI) were recorded to evaluate the severity of inflammation [22]. After 7 consecutive days, all mice were euthanized.

### Histopathological examination

After euthanizing the mice, the back skin samples from each experimental group were fixed with 10% formalin, embedded with paraffin, sectioned, stained with haematoxylin and eosin (HE) and evaluated through light microscope by well-trained pathologists in a double-blind fashion.

### Preparation of splenic single-cell suspension and isolation of CD4^+^ T cells

The spleen tissues of each experimental mouse were fragmented into small pieces and pressed against a 70 μm nylon mesh to obtain splenic single-cell suspension. In addition, the prepared splenic single-cell suspension from 8 IMQ-treated mice was separately isolated by untouched selection method using mouse CD4^+^ T cell isolation kit (MACS, Miltenyi Biotec, Germany). The purity of the cells was 94.13 ± 1.34% as confirmed by flow cytometry analysis, and the vitality was 95.15 ± 1.55% as assessed by trypan blue staining.

### DAPT treatment

Isolated CD4^+^ T cells from IMQ-treated mice were divided into DMSO control group (n = 8) and DAPT-treated groups (each n = 8) at desired concentrations of 2.5, 5, 10, and 20 μmol/l respectively and cultured for 72 h at 37 °C, 5% CO_2_ environment. Then, CD4^+^ T cells were collected for Th17 cell polarization.

### CD4^+^ T cell polarization

1 × 10^6^/ml splenic CD4^+^ T cells were plated in 24-well flat plate (1 ml/cell) and 96-well flat plate (100 μl/cell) in triplicate and polarized under Th17 cell-polarizing circulation consisting of 5 μg/ml CD3 monoclonal antibody (mAb), 2 μg/ml CD28 mAb, 10 ng/ml recombinant IL (rIL)-1β, 20 ng/ml rIL-23, 50 ng/ml rIL-6, 10 μg/ml anti-IL-4 antibody and 10 μg/ml anti-interferon (IFN)-γ antibody. All the antibodies and recombinant cytokines were from eBioscience company (San Diego, CA, USA). Then, cells were collected after 96 h and used for flow cytometric analysis and quantitative real-time RT-PCR detection respectively, and the cell free supernatant was also harvested for cytokine detection by Enzyme-linked immunosorbent assay (ELISA).

### Flow cytometric analysis

For CD4^+^ T cell percentage detection, a total of 1 × 10^6^/ml splenic single-cells were stained with APC-labeled CD3 antibody and FITC-labeled CD4 antibody (eBioscience company) at 4 °C in the dark for 30 min. For Th17 cell percentage analysis (CD4^+^IL17A^+^ T cells/CD4^+^ T cells %), prepared splenic single-cells or CD4^+^ T cells (1 × 10^6^/ml) were firstly stimulated with 25 ng/ml phorbol myristate acetate (PMA, Sigma-Aldrich) and 1 μg/ml ionomycin (Sigma-Aldrich) in the presence of 2 mmol/ml monensin (Sigma-Aldrich) at 37 °C under a 5% CO_2_ environment for 5 h. Next, cells were collected, washed, and surface-stained with FITC-labeled CD4 antibody at 4 °C in the dark for 30 min. Then, cells were fixed, permeabilized, and stained intracellularly with PE-labeled IL-17A antibody (eBioscience company). Flow cytometric analysis was performed on a FACScanto flow cytometer (BD Biosciences company, San Jose, CA, USA). Experiments were performed in triplicate. Isotype controls were used to correct nonspecific binding in all procedures.

### Quantitative real-time RT-PCR analysis of Notch1, Hes-1, RORγt and IL-17A mRNA expression

RNA was extracted by Trizol (Invitrogen, Carlsbad, CA, USA) according to the manufacturer’s instruction. Complementary DNA was synthesized by the PrimeScript™ RT reagent kit (Toyobo, Osaka, Japan). The mRNA expression levels of Notch1, Hes-1, RORγt and IL-17A were quantified by SYBR Premix Ex Taq™ II (TaKaRa) on a Rotor-Gene 3000 quantitative PCR machine (Corbett Research, Sydney, Australia). Each sample was conducted in triplicate and the results were analyzed by the relative standard curve method. The given primers for Notch1, Hes-1, RORγt, IL-17A and β-actin was used as an internal control, were shown in Table 1.

**Table 1:** Primer sequences for Notch1, Hes-1, IL-17A, RORγt and β-actin

Name	Primer sequence	Length of product (bp)
Notch1	5′-TGCCAGTATGATGTGGATGAG-3′	111
	5′-GGTCCCTGTGTAACCTTCTGT-3′	
Hes-1	5′-AGCCCACCTCTCTCTTCTGA -3′	187
	5′-AGGCGCAATCCAATATGAAC-3′	
IL-17A	5′-TTTAACTCCCTTGGCGCAAAA-3′	165
	5′-CTTTCCCTCCGCATTGACAC-3′	
RORγt	5′-AGTGTAATGTGGCCTACTCCT-3′	198
	5′-GCTGCTGTTGCAGTTGTTTCT-3′	
β-actin	5′-CCAGCCTTCCTTCTTGGGTAT-3′	102
	5′-TTGGCATAGAGGTCTTTACGG-3′	

### ELISA analysis of IL-17A concentrations in serum and cell free supernatant

Serum samples of experimental mice, collected and isolated by cardiac puncture, as well as cell free supernatant from DAPT-treated and Th17-polarized splenic CD4^+^ T cells of IMQ-induced mice, were acquired and kept at − 80 °C for further IL-17A concentration analysis by ELISA kit in triplicate (R&D Systems, Minneapolis, MN, USA).

### Statistical analysis

All data were expressed as Mean ± SD. Differences between groups were analyzed by independent-samples *t*-tests and one-way analysis of variance using SPSS 17.0 software. *P* < 0.05 was considered statistically significant.

## Results

### DAPT alleviates the severity of IMQ-induced psoriasis-like skin inflammation

**Fig. 1:**
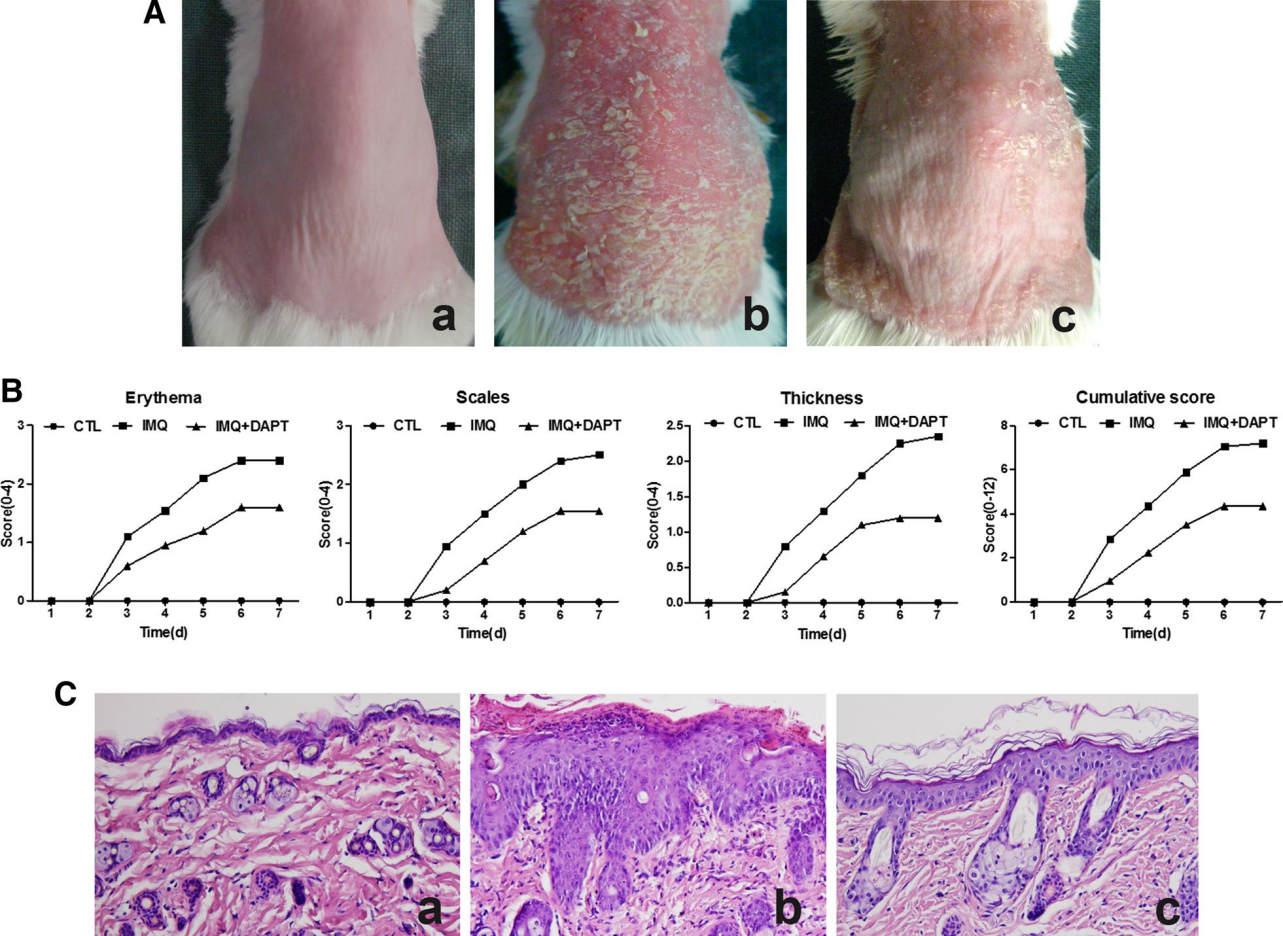
γ-secretase inhibitor DAPT Alleviates the Severity of IMQ-Induced Psoriasis-like Skin Inflammation BALB/c mice were treated daily with IMQ cream (IMQ-treated and IMQ + DAPT-treated groups) or control cream (vaseline group) applied on their shaved back skin. In IMQ + DAPT-treated group, in addition to the topical IMQ application, the mice were co-treated daily with intraperitoneal injection of γ-secretase inhibitor DAPT (10 mg/kg/day). In control and IMQ-treated mice, they received an equal amount of DMSO. After 7 consecutive days of experimental process, mice were euthanized. **A** Phenotypical presentation of mouse back skin after 7 days of treatment. Control mice (a) did not present any sign of inflammation. IMQ-treated mice (b) presented significant inflammation changes of erythema, scaling and thickening. The severity of inflammation was obviously alleviated in IMQ + DAPT-treated mice (c). **B** Erythema, scale, and thickness were scored daily on a scale from 0 to 4: 0, none; 1, slight; 2, moderate; 3, marked; 4, very marked. The cumulative score was calculated by scores of erythema plus scale plus thickness (scale 0–12). Each score of IMQ-treated mice was higher than that of control mice (^#^*P *< 0.01), while, after DAPT and IMQ co-treatment, all of these scores were reduced (^#^*P *< 0.01). **C** Pathological examination of mouse back skin after 7 days of treatment observed by haematoxylin and eosin (HE) stain. The epidermis layer of control mice (a) was thin and composed by only 1–2 layers of epidermal cells. IMQ-treated mice (b) presented obviously epidermal hyperplasia with hyperkeratosis, parakeratosis, Munro microabscess and elongation of trochanterellus as well as massive dermal infiltration of inflammatory cells with dilated capillaries. The severity of epidermal hyperplasia and dermal inflammatory cells infiltration were significantly reduced in IMQ + DAPT-treated mice (c)

To evaluate the alleviatory effect of DAPT on IMQ induced psoriasis-like skin inflammation, IMQ cream was applied on the shaved back skin of BALB/c mice for 7 consecutive days with or without a daily intraperitoneal injection of 10 mg/kg/day DAPT. The control mice did not present any sign of inflammation. 2 or 3 days after IMQ application, the back skin of IMQ-treated mice began to display the signs of psoriasis-like inflammation, such as erythema, scaling and thickening, and the severity of inflammation continually increased up to the end of the experiment. Similar trends in skin inflammation were observed in mice received combined-treatment with IMQ and DAPT, but the symptoms were significantly milder than in mice treated with IMQ alone. The representative examples of phenotypic presentation of mouse back skin in each group after 7 days of treatment and objective scores were shown in Fig. 1A and B respectively. HE-stained skin tissue (Fig. 1C) showed that the epidermis layer of control mice was thin, which was composed by only 1–2 layers of epidermal cells. IMQ-treated mice presented obviously epidermal hyperplasia with hyperkeratosis, parakeratosis, Munro microabscess and elongation of trochanterellus, as well as massive inflammatory cells infiltration in the dermis and dilated capillaries can also be found, which is similar to the typical pathological changes of human psoriasis. While, compared to IMQ-treated mice, both the degrees of epidermal hyperplasia and inflammatory cells infiltration in the dermis were significantly alleviated in mice treated by IMQ and DAPT combinedly.

### DAPT reduces IMQ-induced splenomegaly

**Fig. 2:**
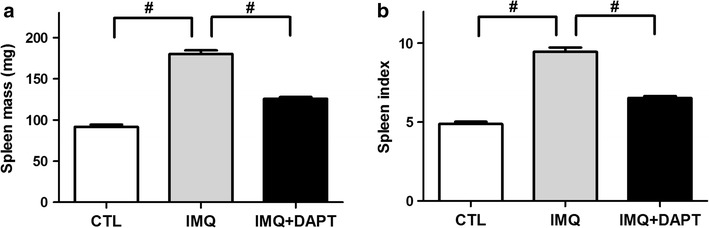
DAPT reduces IMQ-induced splenomegaly mice were euthanized after 7 consecutive days of experimental process, and the spleen mass (**a**) and spleen index (**b**) were observed. Compared to control mice, the spleen mass and spleen index of IMQ-treated mice were significant increased (^#^*P *< 0.01), while combined treatment of DAPT and IMQ led to the reduction of spleen mass and spleen index to a great degree (^#^*P *< 0.01)

Compared to control mice, spleens of IMQ-treated mice were significantly enlarged in weight (179.55 ± 10.78 mg vs. 91.77 ± 8.09 mg, t = 20.591, *P *< 0.01, Fig. 2a) and the spleen index was also obviously increased (9.51 ± 0.49 mg vs. 4.88 ± 0.45, t = 22.144, *P *< 0.01, Fig. 2b). However, DAPT treatment resulted in decreased splenomegaly (124.87 ± 10.50 mg, *P *< 0.01 compared to IMQ-treated mice, Fig. 2a) and spleen index (6.51 ± 0.61, *P *< 0.01 compared to IMQ-treated mice, Fig. 2b) in IMQ + DAPT-treated mice.

### DAPT impairs mRNA expression of Notch1 and its target gene Hes-1

**Fig. 3:**
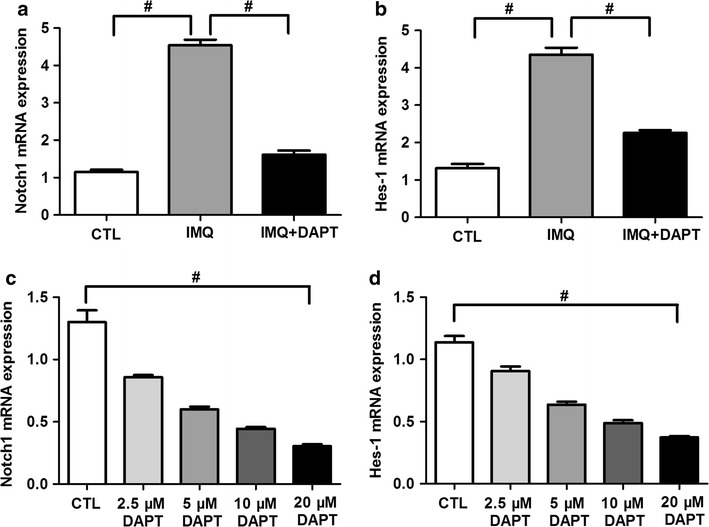
γ-secretase inhibitor DAPT decrease the mRNA expression of Notch1 and its target gene Hes-1. Mice were euthanized after 7 consecutive days of experimental process, and RNA samples from splenic single-cells was used for detection. Both the mRNA expression of Notch1 (**a**) and Hes-1 (**b**) was obviously enhanced in IMQ-treated mice compared to control mice (^#^*P *< 0.01). In IMQ + DAPT-treated mice, Notch1 and Hes-1 mRNA expression was significant decrease (^#^*P *< 0.01). Splenic CD4^+^ T cells from 8 IMQ-treated mice were collected and treated with DAPT, Notch1 (**c**) and Hes-1 (**d**) mRNA expression presented a dramatically reduced trend with the increased concentration of DAPT (^#^*P *< 0.01)

Compared to control mice, IMQ-treated mice showed obviously enhanced Notch1 mRNA expression (4.54 ± 0.0.47 vs. 1.15 ± 0.18, *P *< 0.01, Fig. 3a). DAPT treatment can lead to a distinct reduction of Notch1 mRNA expression in IMQ + DAPT-treated mice (1.62 ± 0.34, *P *< 0.01 comparing to IMQ-treated mice, Fig. 3a). Consistent with Notch1 mRNA expression, IMQ-treated mice also presented increased mRNA expression of its target gene Hes-1 as compared to control mice (4.35 ± 0.57 vs. 1.31 ± 0.36, *P *< 0.01, Fig. 3b), and DAPT treatment can significantly decrease Hes-1 expression (2.26 ± 0.23, *P *< 0.01 comparing to IMQ-treated mice, Fig. 3b). In splenic CD4^+^ T cells from IMQ-treated mice, the mRNA expression of Notch1 and Hes-1 was obviously decreased in DAPT-treated groups compared to control group in a dose-dependent matter (Notch1 mRNA F = 76.471, *P *< 0.01, Fig. 3c; Hes-1 mRNA F = 98.638, *P *< 0.01, Fig. 3d).

### DAPT decreases Th17 cell percentage

**Fig. 4:**
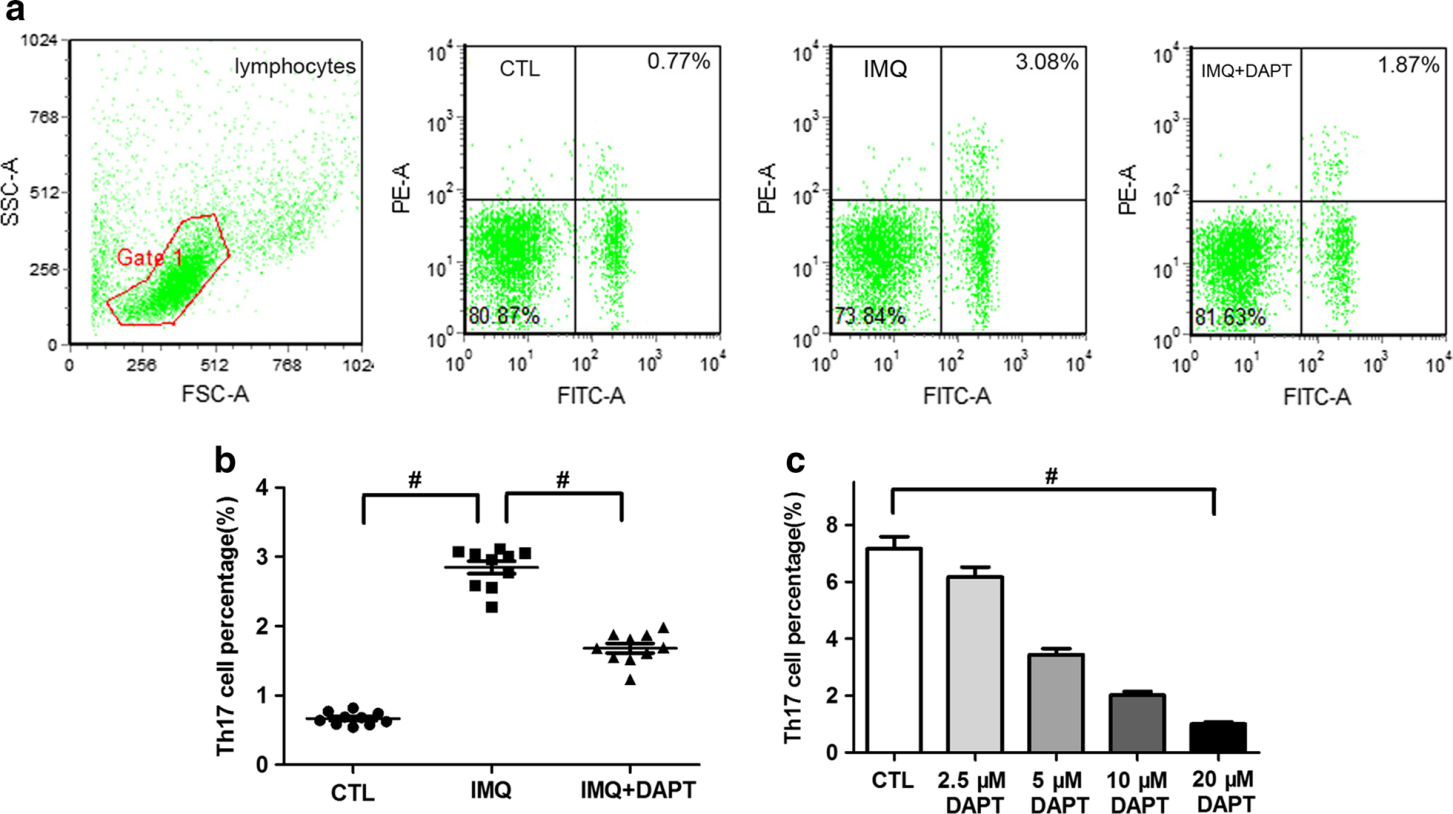
γ-secretase inhibitor DAPT down-regulates the percentage of Th17 cells. Mice were euthanized after 7 consecutive days of experimental process, and percentages of splenic Th17 cells were determined. **a** Representative graphs of flow cytometric analysis of Th17 cell percentage (CD4 was labeled by FITC and IL-17A was labeled by PE) in control mice, IMQ-treated mice and IMQ + DAPT-treated mice. **b** Th17 cell percentage of IMQ-treated mice was obviously enhanced compared to control mice (^#^*P *< 0.01), while the percentage was significantly reduced in IMQ and DAPT combinedly treated experimental mice (^#^*P *< 0.01). **c** After interruption by DAPT in vitro, splenic CD4^+^ T cells of IMQ-treated mice was polarized and detected the change of Th17 cell percentage, which was gradually down-regulated in a dose-dependent matter of DAPT (^#^*P *< 0.01)

Splenic CD4^+^ T cell percentages of control-, IMQ-treated and IMQ + DAPT-treated mice were 15.31 ± 1.78%, 7.99 ± 1.55% and 10.46 ± 1.81% respectively. The representative graphs of flow cytometric analysis of Th17 cell percentage in control-, IMQ-treated and IMQ + DAPT-treated mice were shown in Fig. 4a. Compared to control mice, IMQ-treated mice presented significantly increased Th17 cell percentage (2.85 ± 0.28% vs. 0.67 ± 0.09%, *P *< 0.01, Fig. 4b), while the co-treatment of IMQ and DAPT resulted in decreased proportion of Th17 cells in IMQ + DAPT treated mice (1.68 ± 0.22%, *P *< 0.01 compared to IMQ-treated mice, Fig. 4b). On the other hand, after DAPT treatment in vitro, Th17 cell percentage of splenic CD4^+^ T cells from IMQ-induced mice was obviously decreased in a dose-related fashion with DAPT concentration (F = 107.231, *P *< 0.01, Fig. 4c).

### DAPT reduces mRNA expression and production of Th17 cell effective cytokine IL-17A

**Fig. 5:**
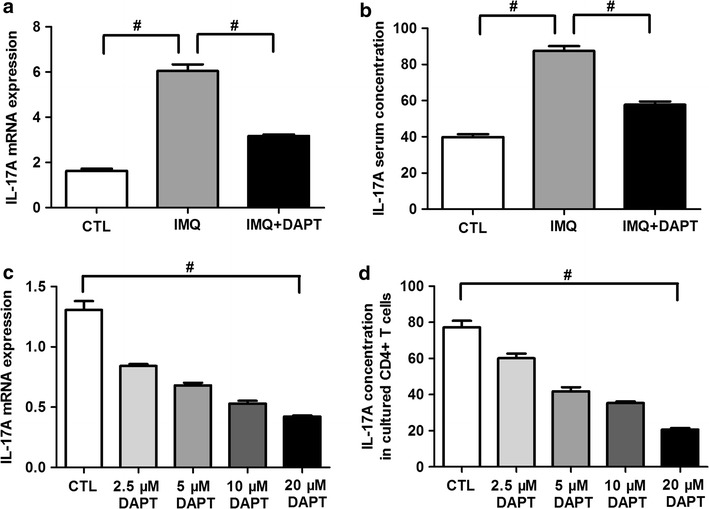
γ-secretase inhibitor DAPT reduces the expression and secretion of IL-17A. Both IL-17A mRNA expression level (**a**) and serum concentration (**b**) in IMQ-treated mice were significantly elevated in comparison with control mice (^#^*P *< 0.01), while the IL-17A expression levels were reduced in IMQ + DAPT-treated mice (^#^*P *< 0.01). After DAPT treatment and Th17-cell polarization in vitro, IL-17A mRNA expression in splenic CD4^+^ T cells (**c**) and its concentration in cell-free supernatant (**d**) of IMQ-treated mice were decreased in DAPT treated groups compared to control group in a dose-dependent way (^#^*P *< 0.01)

IL-17A mRNA expression in IMQ-treated mice were significantly higher than that of control mice (6.05 ± 0.92 vs. 1.63 ± 0.31, *P *< 0.01, Fig. 5a), and DAPT combined treatment dramatically reduced its mRNA expression in IMQ + DAPT-treated mice (3.17 ± 0.23, *P *< 0.01 compared to IMQ-treated mice, Fig. 5a). Further, IL-17A serum concentration of IMQ-treated mice, control mice and IMQ + DAPT-treated mice also presented similar trend (87.48 ± 8.35 pg/ml vs. 39.86 ± 4.69 pg/ml vs. 57.74 ± 5.65 pg/ml, *P *< 0.01, Fig. 5b). In consistent with the change of Th17 cell percentage, IL-17A mRNA expression and concentrations in splenic CD4^+^ T cells and cell free supernatant of IMQ-induced mice were significantly reduced in DAPT-treated groups with a dose-related way (IL-17A mRNA F = 86.957, *P *< 0.01, Fig. 5c; IL-17A concentration F = 101.155, *P *< 0.01, Fig. 5d).

### DAPT Down-regulates mRNA Expression of Th17 Cell Specific Transcription Factor RORγt

**Fig. 6:**
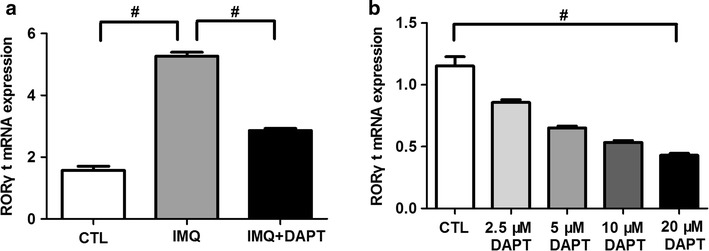
γ-secretase inhibitor DAPT inhibits the expression of RORγt mRNA. **a** RORγt mRNA expression in splenic single-cell suspension was significantly elevated in IMQ-treated mice compared to control mice (^#^*P *< 0.01), while it was obviously decreased in IMQ + DAPT-treated mice (^#^*P *< 0.01). **b** After DAPT treatment and Th17-cell polarization in vitro, RORγt mRNA expression was significantly decreased with the increased dose of DAPT in splenic CD4^+^ T cells of IMQ-treated mice (^#^*P *< 0.01)

To further determine whether the decreased Th17 cell percentage and IL-17A level in the presence of DAPT was correlated with RORγt, the mRNA expression of RORγt was detected and analyzed in vivo and in vitro. Compared to control mice, RORγt mRNA expression presented a clearly elevated level in IMQ-treated mice (5.27 ± 0.38 vs. 1.28 ± 0.27, *P *< 0.01, Fig. 6a), while after interruption by DAPT, there was significant down-regulation of RORγt mRNA expression in IMQ + DAPT-treated mice (2.86 ± 0.21, *P *< 0.01 compared to IMQ-treated mice, Fig. 6a). In vitro study also showed a dose-dependent inhibitory effect of DAPT on RORγt mRNA expression in splenic CD4^+^ T cells of IMQ-induced mice (F = 65.375, *P *< 0.01, Fig. 6b).

## Discussion

Up-regulated expression of Notch1 has been demonstrated in human psoriatic lesions [21]. In this study, we found substantial increase in splenic mRNA expression of Notch1 and its target gene Hes-1 in IMQ-induced mouse psoriasis-like skin inflammation, which closely resembles human psoriasis, presenting as erythema, scaling and thickening in phenotypical characters as well as obviously epidermal hyperplasia, massive inflammatory cells infiltration and capillary dilation in pathological changes. Combined treatment with IMQ topical application and DAPT intraperitoneal injection effectively reduced the severity of erythema, scaling and thickening as well as the epidermal hyperplasia and dermal inflammatory cells infiltration. The splenic mRNA expression of Notch1 and Hes-1 was also significantly decreased after DAPT and IMQ co-treatment. So, our finding indicates that blocking Notch1 signaling by DAPT treatment may contribute to the alleviation of IMQ-induced mouse psoriasis-like skin inflammation.

Markedly elevated expression of Th17 cells and IL-17A in psoriatic lesion and peripheral circulation as well as their positive association with the disease severity have been reported in numerous studies [8, 12–15]. In patients with psoriasis, amelioration of psoriasis is associated with reduced Th17 responses [23–25]. IL-17A, the most important effective cytokine of Th17 cells, which has broad inflammatory effects on keratinocytes and a variety of immune cells found in the skin [9]. Increased levels of IL-17A produce a feed forward inflammatory response in keratinocytes that is self-amplifying and drives the development of mature psoriatic plaques by inducing epidermal hyperplasia, epidermal cell proliferation, and recruitment of leukocyte subsets into the skin [9]. IL-23, a cytokine driving Th17 cell development, is functionally involved in the pathogenesis of psoriasis. The crucial role of IL-23/IL-17 axis in psoriasis has also been demonstrated in mouse psoriasis-like skin inflammation induced by IMQ for its increased Th17 responses both in skin lesion and spleen [22]. Notch1 signaling is critical for the differentiation of Th17 cells, and is activated in vitro-polarized Th17 cell environment of mouse and human [20]. Blockade of Notch1 signaling can result in the dramatic down-regulation of IL-17A and the prevention of Th17-mediated disease progression [20]. In this study, we found that Notch1 inhibition by DAPT can obviously reduce splenic Th17 cell population, mRNA expression of Th17 cell specific transcription factor RORγt, as well as IL-17A mRNA expression and serum concentration in the mouse model of psoriasis-like skin inflammation. In addition, after DAPT treatment, splenic CD4^+^ T cells of IMQ-induced mice presented decreased expression of Th17 cell frequency, RORγt and IL-17A mRNA, and IL-17A secretion in a dose-dependent way in vitro. So, all the data suggest that Th17 cells may be an effective regulating target of Notch1 signaling in psoriasis-like skin inflammation. Notch signaling activation is dependent on γ-secretase [26]. DAPT is a γ-secretase inhibitor and commonly used to block Notch signaling, which can effectively interrupt presenilin, an important component of the γ-secretase and critical for γ-secretase activity, and finally result in the inhibition of active NICD release [27, 28]. Evidences from in vitro study showed that both mouse and human RORγt expression can be significantly reduced by γ-secretase inhibitor treatment and/or Notch1-specific siRNA [20]. RORγt and IL-17 gene promoters are direct transcriptional Notch targets [20, 29]. In addition, along with the alleviation of psoriatic lesions by ustekinumab treatment, a mAb against the p40 subunit of IL-23, the protein expression levels of Notch1 and Hes-1 from ustekinumab-treated psoriatic samples were dramatically decreased compared to untreated psoriatic skin [30]. So the above research reports and our findings in the present study corroborate our suggestion that inhibition of Notch1 signaling is a potential therapeutic target of psoriatic inflammation for its regulating effect on Th17 responses.

Psoriasis is a immune-mediated, systemic inflammatory disease [3, 4]. Dramatically increased percentage of Th17 cells has been demonstrated in human psoriatic lesion, more importantly, it is twofold or even higher fold than Th17 cell proportion in peripheral circulation [7, 8]. Moreover, most lesional Th17 cells can produce IL-17A in vitro without further stimulation [7]. Besides Th17 cells, other types of IL-17 producing cells have been found in the skin, including CD8^+^ T cells (Tc17), innate lymphoid cells, and γδT cells [31–33]. γδT cells have been reported to be a major IL-17 producers in human psoriatic lesion, which are also stimulated and regulated by IL-23 as well as largely dependent on RORγt [34–36]. In IMQ-treated mice, γδT cells were increased in both dermal and lymph nodes and secreted large amount of IL-17, while the frequency of splenic γδT cells and IL-17 production from γδT cells were not altered, which is consistent with the IL-23-injected psoriasis model [34]. Interestingly, the number of splenic Th17 cells is increased in the same mouse psoriatic model induced by IMQ [22]. So Th17 cells and γδT cells may corporately contribute to the production and effect of IL-17 in psoriatic lesion, while to a large extent, the increased amount of IL-17 in peripheral circulation, may be attributed to Th17 cells. Notch1-Hes1 signaling has been reported to be critical for the development of IL-17-producing γδ T cells, and conditional Hes-1 ablation in peripheral γδT cells decreases their IL-17 production [36]. Base on the above researches, it is tempting to speculate that inhibition of Notch1 signaling by DAPT may also contribute to the decrease of IL-17 produced by γδ T cells and the alleviation of psoriatic inflammation caused by γδ T cells, which needs to be confirmed in further research.

IMQ is a ligand for TLR7 and TLR8, and a potent immune activator [37]. IMQ can induce exacerbation of psoriasis both at treated lesion and even at distant skin sites previously unaffected [38–40]. Important hallmarks of IMQ induced psoriasis are the infiltration of plasmacytoid dendritic cells (pDC) and type I IFN activity [37]. Accordingly, IMQ application on mouse skin leads to rapid influx of pDC [41]. pDCs also play a critical role in the pathogenesis of SLE through IFN-α production upon TLR-7/TLR-9 ligation [42, 43]. Recently, Yokogawa et al. reported that lupus-like systemic autoimmune disease in mice can be induced by 1.25 mg of 5% IMQ cream topical application on ears with 125 µg of IMQ intraperitoneal injection 3 times weekly for a 4-week protocol [44]. And during the experimental setting of lupus-like systemic autoimmune disease, full-blown psoriatic lesions were observed within 3 weeks of IMQ treatment but were attenuated thereafter. Thus, it is likely that the pathogenesis of both psoriasis and lupus share roles of PDCs and type I IFN [44].

## Conclusions

The current study suggests that Notch1 inhibition by DAPT can effectively alleviate the severity of mouse psoriasis-like skin inflammation by regulating the differentiation and function of Th17 cells, indicating that DAPT might be a potential therapeutic candidate for the treatment of psoriatic inflammation.

## Abbreviations

Th: T helper
NICD: Notch receptor intracellular domain
DMSO: dimethylsulfoxide
HE: haematoxylin and eosin
mAb: monoclonal antibody
PMA: phorbol myristate acetate
Th17: IL-17-producing CD4^+^ T helper cells
